# Comparative analysis of housekeeping and tissue-specific driver nodes in human protein interaction networks

**DOI:** 10.1186/s12859-016-1233-0

**Published:** 2016-09-09

**Authors:** Xiao-Fei Zhang, Le Ou-Yang, Dao-Qing Dai, Meng-Yun Wu, Yuan Zhu, Hong Yan

**Affiliations:** 1 School of Mathematics and Statistics & Hubei Key Laboratory of Mathematical Sciences, Central China Normal University, Luoyu Road, Wuhan, 430079 China; 2 College of Information Engineering, Shenzhen University, Nanhai Ave 3688, Shenzhen, 518060 China; 3 Intelligent Data Center and Department of Mathematics, Sun Yat-Sen University, Xingang West Road, Guangzhou, 510275 China; 4 School of Statistics and Management, Shanghai University of Finance and Economics, Guoding Road, Shanghai, 200433 China; 5 School of Automation, China University of Geosciences, Lumo Road, Wuhan, 430074 China; 6 Department of Electronic and Engineering, City University of Hong Kong, Tat Chee Avenue, Hong Kong, China

**Keywords:** Protein interaction network, Driver proteins, Tissue specificity, Minimum dominating set, Collective influence

## Abstract

**Background:**

Several recent studies have used the Minimum Dominating Set (MDS) model to identify driver nodes, which provide the control of the underlying networks, in protein interaction networks. There may exist multiple MDS configurations in a given network, thus it is difficult to determine which one represents the real set of driver nodes. Because these previous studies only focus on static networks and ignore the contextual information on particular tissues, their findings could be insufficient or even be misleading.

**Results:**

In this study, we develop a Collective-Influence-corrected Minimum Dominating Set (CI-MDS) model which takes into account the collective influence of proteins. By integrating molecular expression profiles and static protein interactions, 16 tissue-specific networks are established as well. We then apply the CI-MDS model to each tissue-specific network to detect MDS proteins. It generates almost the same MDSs when it is solved using different optimization algorithms. In addition, we classify MDS proteins into Tissue-Specific MDS (TS-MDS) proteins and HouseKeeping MDS (HK-MDS) proteins based on the number of tissues in which they are expressed and identified as MDS proteins. Notably, we find that TS-MDS proteins and HK-MDS proteins have significantly different topological and functional properties. HK-MDS proteins are more central in protein interaction networks, associated with more functions, evolving more slowly and subjected to a greater number of post-translational modifications than TS-MDS proteins. Unlike TS-MDS proteins, HK-MDS proteins significantly correspond to essential genes, ageing genes, virus-targeted proteins, transcription factors and protein kinases. Moreover, we find that besides HK-MDS proteins, many TS-MDS proteins are also linked to disease related genes, suggesting the tissue specificity of human diseases. Furthermore, functional enrichment analysis reveals that HK-MDS proteins carry out universally necessary biological processes and TS-MDS proteins usually involve in tissue-dependent functions.

**Conclusions:**

Our study uncovers key features of TS-MDS proteins and HK-MDS proteins, and is a step forward towards a better understanding of the controllability of human interactomes.

**Electronic supplementary material:**

The online version of this article (doi:10.1186/s12859-016-1233-0) contains supplementary material, which is available to authorized users.

## Background

As chief actors within cells, proteins rarely act alone. Diverse biological processes within cells are carried out by molecular machines which are built from a set of physically interacting proteins [1, 2]. Proteins, together with their interactions, can be modeled as a network, where nodes represent proteins and links represent interactions between proteins. Because of the interactions, perturbations of a specific set of structural nodes can alter the state of the entire network [3–10]. Therefore, identifying the minimal set of driver proteins which can control the entire network has become an important task in network biology [6, 11–13].

Recently, Liu et al. [3] developed a ground-breaking method that identified a minimum set of driver nodes by computing a maximum bipartite matching. However, their method can only be applied to directed networks. Nacher and Akutsu [14] developed an equivalent optimization model from the perspective of Minimum Dominating Set (MDS) to analyze undirected networks [15]. For convenience, we refer to their model as standard MDS model. In a protein interaction network, an MDS is defined as an optimized subset of proteins where each Non-MDS (NMDS) protein is adjacent to an element of MDS [6]. Several recent studies applied the standard MDS model to protein interaction networks and found that MDS proteins were not only located in central network positions but also enriched with important biological functions and features [6, 11–13]. The topological and functional significance of MDS proteins demonstrate the importance of MDS model in providing new views of structural controllability of protein interaction networks.

There may exist multiple MDS configurations for a given network [16]. The different optimization algorithms used to solve the standard MDS model may produce quite different configurations. Thus, it is difficult to determine which one is the real set of nodes that can control the entire network [12, 16]. Furthermore, previous studies on network controllability just focus on static networks without any information about where and when each interaction occurs. Within a particular tissue, only a subset of proteins can be expressed and only the interactions between those expressed proteins can occur [17, 18]. Consequently, results obtained from static networks without information of tissue specificity could be insufficient and even be misleading.

Several high-throughput experimental technologies have been developed to map out which proteins are expressed in particular tissues [19–26]. With the availability of large-scale tissue expression data for human, tissue-specific protein interaction networks can be constructed by integrating molecular expression data with static protein interaction data [27–30]. Based on these constructed tissue-specific networks, several studies found that tissue-specific proteins and housekeeping proteins had distinct topological and functional properties [31–34]. Tissue-specific networks have been used to identify drug-targets [31], prioritize disease genes [35–37], and illustrate relationships among diseases [38]. To reveal the biological significance of hub proteins in tissue-specific networks, Kiran and Nagarajaram [39] classified hub proteins into tissue-specific hubs and housekeeping hubs. Comparison between these two categories of hubs showed that they exhibited distinct properties. These studies on the construction and application of tissue-specific networks motivate us to identify driver nodes in tissue-specific networks and to explore their topological and functional significance.

In this study, we integrate diverse genome-scale data to construct tissue-specific protein interaction networks. In addition, we propose a Collective-Influence-corrected MDS (CI-MDS) model by extending the standard MDS model to capture heterogeneity in collective influence [7, 8] of proteins. The proposed model can significantly improve the overlap between the sets of MDS proteins calculated by different optimization algorithms. We apply the CI-MDS model to each tissue-specific network to identify MDS proteins and then classify the detected MDS proteins into Tissue-Specific MDS (TS-MDS) proteins and HouseKeeping MDS (HK-MDS) proteins. Experiment results show that TS-MDS proteins and HK-MDS proteins have significantly different topological and functional characteristics. Our study exposes distinct properties of MDS proteins involved in tissue-specific networks, suggesting that tissue specificity is important in studying the controllability of protein interaction networks.

## Results

### Construction of tissue-specific networks

**Fig. 1:**
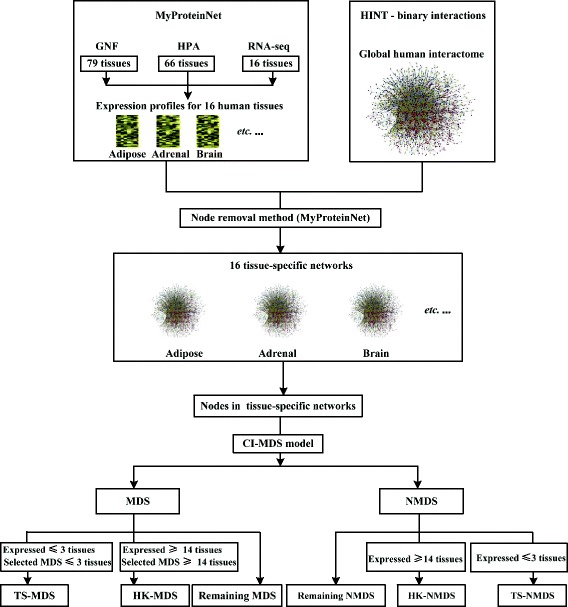
The construction of tissue-specific networks and the classification of MDS proteins. Following the method of [42], molecular expression profiles across 16 human tissues are obtained by consolidating three types of data (GNF [20], HPA [24] and RNA-seq [41]). In parallel, high-quality binary interactions for H. sapiens are collected from the HINT database [40]. Tissue-specific networks are constructed by removing proteins that are not expressed in the corresponding tissues from the global network. Then the CI-MDS model is applied to each tissue-specific network and proteins are classified into MDS proteins and Non-MDS (NMDS) proteins. MDS proteins are further categorized into HouseKeeping MDS (HK-MDS) proteins and Tissue-Specific MDS (TS-MDS) proteins based on the number of tissues in which they are expressed and identified as MDS proteins

We collect high-quality binary interactions for human from the High-quality INTeractomes (HINT) database [40]. The resulting network, which consists of 56,695 interactions between 12,539 proteins, is referred to as global interaction network (Fig. 1). In parallel, we consider tissue-specific expression profiles in the MyProteinNet database [29] which are collected from three major resources: (1) the Genomics Institute of the Novartis Research Foundation (GNF) dataset based on profiling using DNA microarrays [20], (2) the Human Protein Atlas (HPA) dataset based on protein immunohistochemistry measurements [24], (3) the Illumina Body Map 2.0 dataset based on RNA-seq measurements [41]. The three datasets contain expression profiles across 79, 66 and 16 human tissues, and here we only consider the 16 main tissues which are shared by the three datasets [27, 29]. For each expression data, determining whether a gene is expressed in a tissue is done using stringent thresholds (see “Methods” for details). A gene is considered to be expressed in a tissue if it is found to be expressed in that tissue according to at least one expression data.

We integrate tissue-specific expression profiles and global interaction network to construct tissue-specific networks following the method of node removal [36, 42] (Fig. 1). Specially, a tissue-specific network is constructed by removing proteins that are not expressed in the tissue from the global network. That is, each tissue-specific network only contain interactions between proteins that are expressed in this tissue simultaneously. We implement this method using the MyProteinNet database [29] which is developed for building tissue-specific networks by filtering a global interactome in terms of tissue-specific expression data. In our experiments, we use the default expression thresholds provided in MyProteinNet. To remove isolated interactions that significantly affect the identified driver nodes, we only consider the largest connected component of each tissue-specific network. The constructed tissue-specific networks are available in Additional file 1.

**Fig. 2:**
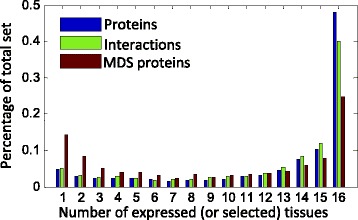
The distribution of proteins, interactions and MDS proteins across 16 tissues. For proteins and interactions, the *x-axis* denotes the number of tissue in which they are expressed; for MDS proteins, the x-axis denotes the number of tissue in which they are identified as MDS proteins. The *y-axis* denotes the frequency. The distribution of proteins, interactions and MDS proteins by the number of tissues in which they are expressed (or selected as MDS proteins) is bi-modal, with most of them being globally (14 – 16 tissues) or tissue-specific (1 – 3 tissues)

We find that 42,290 interactions involving 9834 proteins can occur in at least one of the 16 main tissues, and each tissue-specific network covers only a part of proteins (66.51 – 89.06 %) and interactions (61.45 – 88.41 %) (Table 1). We also observe a bi-modal distribution of expressed proteins across tissues (Fig. 2): 65.9 % of proteins are expressed in 14 – 16 tissues (housekeeping proteins), and 10.7 % of proteins are expressed in 1 – 3 tissues (tissue-specific proteins), which is in agreement with previous observations [42]. Several studies have performed a comprehensive analysis of housekeeping proteins and tissue-specific proteins [31, 32, 34, 42]. Thus, we do not repeat the analysis below.

**Table 1:** Statistics of tissue-specific networks and their corresponding MDS proteins

Tissue	No. (pct) of proteins	No. (pct) of interactions	No. (pct) of MDS proteins
Adipose	6,751 (68.65 %)	26,813 (63.40 %)	1,170 (17.33 %)
Adrenal	8,172 (83.10 %)	33,982 (80.35 %)	1,370 (16.76 %)
Brain	8,239 (83.78 %)	34,223 (80.92 %)	1,394 (16.92 %)
Breast	7,836 (79.68 %)	32,839 (77.65 %)	1,318 (16.82 %)
Colon	7,875 (80.08 %)	32,641 (77.18 %)	1,335 (16.95 %)
Heart	7,669 (77.98 %)	31,471 (74.42 %)	1,303 (16.99 %)
Kidney	8,162 (83.00 %)	33,888 (80.13 %)	1,384 (16.96 %)
Liver	7,148 (72.69 %)	28,553 (67.52 %)	1,250 (17.49 %)
Lung	8,319 (84.59 %)	35,240 (83.33 %)	1,412 (16.97 %)
Lymph node	8,036 (81.72 %)	33,804 (79.93 %)	1,361 (16.94 %)
Ovary	7,839 (79.71 %)	32,450 (76.73 %)	1,311 (16.72 %)
Prostate	8,162 (83.00 %)	34,349 (81.22 %)	1,370 (16.79 %)
Skeletal muscle	7,032 (71.51 %)	28,151 (66.57 %)	1,213 (17.25 %)
Testis	8,758 (89.06 %)	37,388 (88.41 %)	1,455 (16.61 %)
Thyroid	8,098 (82.35 %)	34,024 (80.45 %)	1,358 (16.77 %)
White blood cells	6,541 (66.51 %)	25,986 (61.45 %)	1,131 (17.29 %)

### Determination of MDS proteins in tissue-specific networks

**Fig. 3:**
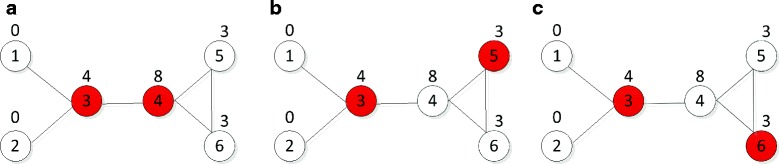
A graphical example that illustrates the CI-MDS model. A minimum dominating set (MDS) is defined as an optimized subset of proteins (*red* nodes) from which each remaining (i.e., NMDS) protein (*white* nodes) can be reached by at least one interaction. For the given toy network, there exists three different MDS configurations : (**a**) {3, 4}, (**b**) {3, 5} and (**c**) {3, 6}. Therefore, it is difficult to determine which one is the real set of controller nodes according to the standard MDS model. To overcome this problem, we introduce a CI-MDS model which takes into account the collective influence of proteins. Here we compute the collective influence of each protein with *ℓ*=1 (*above* the nodes). The collective influence of protein 4 is higher than those of proteins 5 and 6. According to the CI-MDS model, proteins {3, 4} are determined as an optimal MDS because its members have the highest collective influence among all the three possible MDS configurations

In a protein interaction network, we define a Minimum Dominating Set (MDS) as the smallest subset of proteins from which each Non-MDS (NMDS) protein can be reached by one interaction (Fig. 3) (see “Methods”). In other words, each NMDS protein must be connected to at least one MDS protein. As mentioned in [12, 16], there may exist more than one MDS configuration in a given network (Fig. 3). Therefore, different results may be generated by using different optimization algorithms to solve the standard MDS model [6, 14]. To overcome this problem, we develop a Collective-Influence-corrected Minimum Dominating Set (CI-MDS) model by taking into account the collective influence of proteins (see “Methods”). We apply the standard MDS model and the CI-MDS model on each tissue-specific network to detect tissue-dependent MDS proteins. We solve the two models by using two different optimization methods: “lp_solve” [43] and “intlinprog” [44]. There is a distance parameter *ℓ* in the proposed CI-MDS model. To investigate the effect of *ℓ*, we try several different values (e.g., *ℓ*=0,1,2,3). The standard MDS model produces quite different MDSs by using different optimization algorithms, but the CI-MDS model (with *ℓ*≥1) generates almost the same MDSs (Additional file 2).

To investigate the effect of distant parameter *ℓ*, we compute the overlap between MDSs identified by the CI-MDS model with different values of *ℓ*. We find that the overlap between the resulting MDSs is large (Additional file 3), which indicates that the CI-MDS model is not very sensitive to the choice of *ℓ*. In the following experiments, we set *ℓ*=1 for the following reasons: (1) the collective influence with *ℓ*≥1 has a richer topological content than the square of reduced degree (*ℓ*=0) [7], which can be validated by the higher overlap between MDSs calculated using different optimization methods for *ℓ*≥1 (Additional file 2); (2) *ℓ* cannot be too large because the boundary of the network can be reached for large *ℓ*, diminishing collective influence of nodes [7, 8]; (3) when *ℓ*=1,2,3, the overlap between resulting MDSs is large (Additional file 3). In the following text, unless otherwise stated, we mean that MDS proteins are those identified by the CI-MDS model with *ℓ*=1.

Table 1 presents the number (and percentage) of MDS proteins determined in each tissue-specific network. We find that about 17 % of proteins can dominate the entire network for each tissue. We also observe the distribution of MDS proteins across tissues is bi-modal (Fig. 2): 38.5 % of MDS proteins are formed in 14 – 16 tissues, and 27.6 % of MDS proteins are formed in 1 – 3 tissues.

### Determination of housekeeping and tissue-specific MDS proteins

Proteins in tissue-specific networks can be categorized into MDS proteins and NMDS proteins (Fig. 1). A protein is considered to be an MDS protein if it is identified as an MDS protein in at least one tissue-specific network, and it is considered to be a NMDS protein otherwise. Of the 9,834 total proteins, 2,265 are MDS proteins. Proteins are further grouped into six distinct classes in terms of the number of tissues in which they are expressed and selected as MDS proteins: (1) HouseKeeping MDS (HK-MDS): proteins that are expressed in at least 14 tissues and also identified as MDS proteins in at least 14 tissues; (2) Tissue-Specific MDS (TS-MDS): proteins that are expressed in at most 3 tissues and also selected as MDS proteins in those tissues; (3) Remaining MDS: MDS proteins which are neither HK-MDS proteins nor TS-MDS proteins; (4) HouseKeeping Non-MDS (HK-NMDS): NMDS proteins expressed in at least 14 tissues; (5) Tissue-Specific Non-MDS (TS-NMDS): NMDS proteins expressed in at most 3 tissues; (6) Remaining NMDS: NMDS proteins which are neither HK-NMDS proteins nor TS-NMDS proteins. Among the 2,265 MDS proteins, 872 are HK-MDS proteins and 125 are TS-MDS proteins (Additional file 4). Among the 7,569 NMDS proteins, 4,771 are HK-NMDS proteins and 865 are TS-NMDS proteins. Comparative analysis of TS-MDS, HK-MDS and Remaining MDS proteins reveals that TS-MDS proteins and HK-MDS proteins exhibit different properties, as discussed below, while Remaining MDS proteins perform as a trade-off between TS-MDS proteins and HK-MDS proteins. Thus, we mainly focus on comparative analysis of HK-MDS proteins and TS-MDS proteins.

### HK-MDS proteins are more central than TS-MDS proteins in the interactomes

**Fig. 4:**
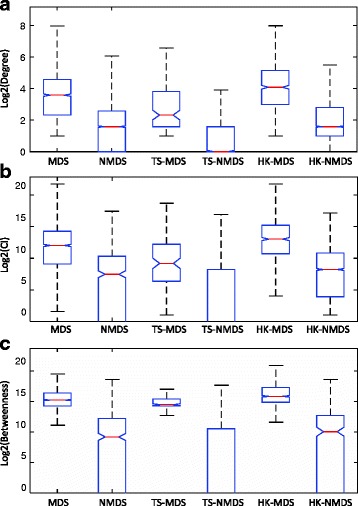
Distribution of (**a**) degree, (**b**) collective influence (*ℓ*=1) and (**c**) betweenness of different types of proteins. The distribution is represented by box plots (*line = median*). In each figure, outliers have been masked for clarity

The centrality-lethality rule demonstrates that there exists a strong correlation between node’s topological centrality and its functional importance in a protein interaction network [11, 45]. We wonder whether there is significant difference between topological centralities of different types of proteins. Three node centralities (degree [46], collective influence [7] and betweenness [47]) are considered. Degree centrality counts the number of interacting partners of the protein, and proteins with high degree are likely to be essential [46]. Collective influence is the product of the protein’s reduced degree and the sum of the reduced degrees of its interacting neighbors (*ℓ*=1) [7]. Proteins with high collective influence are likely to be driver nodes in the network. Betweenness centrality counts the number of shortest paths from all proteins to all others proteins that pass through the protein [47]. A node with high betweenness has a large influence over the “information transfer” [48] and can act as important connectors in the network [49]. The three centralities for each protein are calculated using the global network in this study. From Fig. 4, we find that the degree, collective influence and betweenness of MDS proteins are significantly higher than those of NMDS proteins (Kolmogrov-Smirnov test, Additional file 5). Furthermore, HK-MDS proteins are significantly more topologically central than TS-MDS proteins (Additional file 5).

### HK-MDS proteins perform more biological functions than TS-MDS proteins

**Fig. 5:**
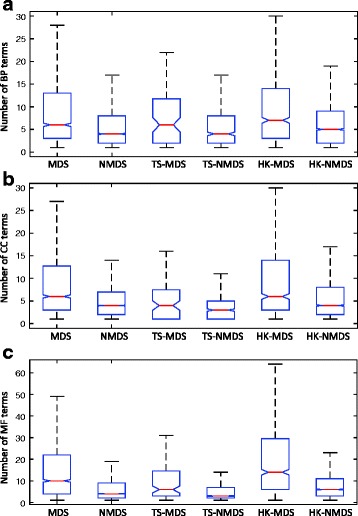
Distribution of the number of associated (**a**) biological process, (**b**) cellular component and (**c**) molecular function terms of different types of proteins. The distribution is represented by box plots (*line = median*). In each figure, outliers have been masked for clarity. Only direct GO annotations are taken into account

Multifunctional proteins often interact with distinct sets of partners to carry out different biological functions [50–53]. Therefore, they may play important roles in cells. We wonder whether different types of proteins are involved in different number of biological functions. For each protein, the number of associated Gene Ontology (GO) terms is calculated by exploring GO annotations [54]. Here we only consider direct GO annotations. All the three domains (Biological Process (BP), Cellular Component (CC) and Molecular Function (MF)) are considered. From Fig. 5, we observe that MDS proteins are significantly associated with more functions than NMDS proteins (Kolmogrov-Smirnov test, Additional file 6). Moveover, HK-MDS proteins carry out more biological roles than TS-MDS proteins. Similar results are observed when we consider both direct GO annotations and all parent terms (Additional files 6 and 7).

### HK-MDS proteins evolve more slowly than TS-MDS proteins

**Fig. 6:**
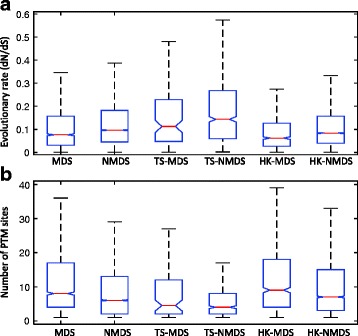
Distribution of (**a**) evolutionary rates and (**b**) number of post-translational modification sites of different types of proteins. The distribution is represented by box plots (*line = median*). In each figure, outliers have been masked for clarity

Evolutionary rates of genes are affected by their essentiality and expression patterns [55], and are negatively correlated with their importance [56]. Previous studies have shown that proteins with many interactions are under evolutionary pressure compared with proteins with a few interactions [57]. Therefore, we would like to investigate the evolutionary rates of different types of proteins. The evolutionary rates of proteins are estimated by employing their dN/dS values obtained from the Ensembl database [58]. MDS proteins, in general, are significantly evolving at slower rates than NMDS proteins (Fig. 6a, Additional file 8). Among MDS proteins, HK-MDS proteins evolve significantly more slowly than TS-MDS proteins.

### HK-MDS proteins have more post-translational modification sites than TS-MDS proteins

Post-Translational Modification (PTM), which mostly occurs on functional domains of proteins, can affect protein conformational and functional specificities [59, 60]. Proteins with high PTMs tend to occupy central positions in the interactions network [60]. Therefore, we wonder whether the distribution of the number of PTM sites of different types of proteins significantly differ. We retrieve the number of PTM sites of proteins from the dbPTM database [61]. Compared with NMDS proteins, MDS proteins have a greater number of PTM sites (Fig. 6b, Additional file 8). Moreover, we find that HK-MDS proteins are subjected to a greater number of PTM sites than TS-MDS proteins.

### HK-MDS proteins are significantly enriched with essential genes

Essential genes are genes that are indispensable for the survival of the organisms [62], therefore they can be considered as one type of human biologically central genes. To reveal the biological significance of different types of MDS proteins, we wonder whether these proteins are significantly enriched with essential genes. Out of the 2,501 essential genes obtained from the Database of Essential Genes (DEG) [62], 1,911 are found in our considered interaction network. Fisher’s exact test is applied to evaluate the statistical significance. We observe that essential genes are significantly enriched in MDS proteins and HK-MDS proteins (*p*-value ≤0.05) (Table 2). Among the total of 2,265 MDS proteins, 638 (28.2 %) are essential genes; while there are 283 (32.5 %) essential genes among 872 HK-MDS proteins. This indicates HK-MDS proteins are more likely to be essential than MDS proteins. In addition, TS-MDS proteins are not significantly enriched with essential genes.

**Table 2:** Biological centrality of different types of MDS proteins

	MDS (2,265)		TS-MDS (125)		HK-MDS (872)	
Biologically central proteins (No.)	No (pct) of BC	*p*-value	No (pct) of BC	*p*-value	No (pct) of BC	*p*-value
Essential genes (1,911)	638 (28.2 %)	5.4E-31	23 (18.4 %)	9.1E-01	283 (32.5 %)	1.0E-21
Ageing genes (267)	140 (6.2 %)	5.0E-26	4 (3.2 %)	5.8E-01	83 (9.5 %)	8.7E-26
Virus-targeted proteins (1,934)	646 (28.5 %)	1.2E-31	21 (16.8 %)	5.0E-01	327 (37.5 %)	6.4E-38
Transcription factors (156)	64 (2.8 %)	4.3E-07	4 (3.2 %)	1.4E-01	38 (4.4 %)	5.4E-09
Protein kinases (392)	146 (6.4 %)	1.2E-10	3 (2.4 %)	4.9E-01	62 (7.1 %)	6.8E-06
Disease-related genes (2,022)	594 (26.2 %)	1.1E-13	45 (36.0 %)	7.5E-05	223 (25.6 %)	1.9E-04
Cancer-related genes (791)	234 (10.3 %)	8.4E-06	17 (13.6 %)	2.9E-02	76 (8.7 %)	4.3E-01

### HK-MDS proteins are significantly enriched with ageing genes

Ageing genes which relate to longevity are biologically central in the process of ageing [63]. To show the biological significance of different types of MDS proteins, we investigate whether ageing genes are significantly enriched in the sets of identified MDS proteins. After retrieving 298 ageing genes from the Aging Gene (GenAge) Database [63], we find that there are 267 ageing genes in our considered interaction network. We apply Fisher’s exact test to evaluate the statistical significance and find that ageing-related genes are indeed significantly enriched in the set of MDS proteins and the set of HK-MDS proteins (Table 2). On the other hand, ageing genes do not significantly appear in the set of TS-MDS proteins.

### HK-MDS proteins are significantly enriched with virus-targeted proteins

Human viruses seize host proteins to control a host cell and cause some diseases [64], suggesting that virus-targeted proteins play functionally central roles in the cells. Therefore, we expect that proteins targeted by viruses may significantly appear in MDS proteins. Out of 2,420 human virus-targeted proteins obtained from the VirusMentha database [65], 1,934 are found in the interaction network. Applying Fisher’s exact test, we find that virus-targeted proteins are significantly enriched in the set of MDS proteins and the set of HK-MDS proteins (Table 2). We also observe that TS-MDS proteins do not significantly enriched with virus-targeted proteins.

### HK-MDS proteins are significantly enriched with transcription factors

Transcription factors are important proteins that govern the expression of their underlying target genes [66]. Assuming that MDS proteins may significantly contribute to control process, we expect that transcription factors may be significantly enriched in the sets of MDS proteins. In particular, we collect 222 transcription factors from the TRANSFAC database [67], and find that 156 proteins belong to our considered interaction network. From Table 2, we observe that transcription factors are indeed significantly enriched in MDS proteins and HK-MDS proteins (Fisher’s exact test). On the other hand, TS-MDS proteins are not significantly enriched with transcription factors.

### HK-MDS proteins are significantly enriched with protein kinases

Protein kinases that control the level of phosphorylation of their substrates play central roles in cellular signalling, metabolism, cellular transport, and many other cellular pathways [68]. To indicate functional significance of MDS proteins, we hypothesize that such sets may be significantly enriched with proteins that govern phosphorylation. Out of 516 human protein kinases from the Regulatory Network in Protein Phosphorylation (RegPhos) database [69], 392 are found in our considered interaction network. We find that protein kinases significantly appear in MDS proteins and HK-MDS proteins (Table 2, Fisher’s exact test). We also observe that TS-MDS proteins are less likely to be kinases.

### Both TS-MDS proteins and HK-MDS proteins are significantly enriched with disease-related genes

Proteins that govern diseases have special biological roles in the cells [70], suggesting that MDS proteins may be significantly enriched with protein associated with diseases. Out of 3,182 disease-related genes retrieved from the Online Mendelian Inheritance in Man (OMIM) database [71], 2,022 belong to the interaction network which we consider. Applying Fisher’s exact test, we find that all the three types of MDS proteins are significantly enriched with disease-related genes (Table 2). Furthermore, TS-MDS proteins are more likely to be associated with diseases than HK-MDS proteins. This may be partly due to tissue-specific manifestation of hereditary diseases [18, 42]. The reason why HK-MDS proteins are also significantly enriched with disease-related genes may be attributed to the fact that most of disease-related genes are widely expressed across tissues [42].

### TS-MDS proteins are significantly enriched with cancer-related genes

Cancer-related genes play a crucial roles in the development and progression of cancer. Therefore, it is interested to analyze whether cancer-related genes are significantly enriched in the sets of MDS proteins. We collect 1,448 cancer-related genes from the Genome-Wide Association Studies (GWAS) Catalo database [72], and there are 791 cancer-related genes in our considered interaction network. According to Fisher’s exact test, we observe that the set of MDS proteins and the set of TS-MDS proteins are significantly enriched with cancer-related genes, while the cancer-related genes do not significantly appear in the set of HK-MDS proteins. This observation is in accord with the common knowledge that tumors are originated from specific organs [73].

### Functional enrichment analysis of TS-MDS proteins and HK-MDS proteins

To compare the biological significance of TS-MDS proteins and HK-MDS proteins, their enrichment in GO terms are computed using DAVID [74]. The three domains, namely, biological process, cellular component, and molecular function are considered. We assume that a set of proteins is significantly associated with a GO term if the p-value is lower than 0.05.

Our GO term enrichment analysis regarding biological process reveals that TS-MDS proteins are mainly involved in tissue-specific processes such as cell-cell signaling, blood circulation, neuron projection development, and feeding behavior, while that HK-MDS proteins are mainly involved in core processes critical for normal cellular functioning such as regulation, protein transport, protein modification, protein localization, complex assembly, and phosphorylation (Table 3, Additional file 9). When considering cellular component, TS-MDS proteins are enriched with GO terms related to plasma membrane, synapse, cell junction, and extracellular region, while HK-MDS proteins are enriched with GO terms related to cytosol, nuclear lumen, organelle lumen, nucleoplasm, transcription factor complex, nucleolus, chromosome, vesicle, and endomembrane system. For the molecular function domain, we find that TS-MDS proteins are primarily enriched in sequence-specific DNA binding, enzyme inhibitor activity, estrogen receptor activity, endopeptidase inhibitor activity, gated channel activity, and calcium ion binding, whereas HK-MDS proteins are primarily enriched in transcription factor binding, identical protein binding, enzyme binding, small conjugating protein ligase activity, protein C-terminus binding, and protein kinase activity. These findings indicate that TS-MDS proteins are mainly responsible for tissue specific functions and HK-MDS proteins are mainly involved in core cellular machineries.

**Table 3:** GO term enrichments for TS-MDS proteins and HK-MDS proteins

	TS-MDS		HK-MDS	
Domains	GO terms	*p*-value	GO terms	*p*-value
BP	Cell-cell signaling	3.7E-05	Regulation of apoptosis	2.4E-18
	Circulatory system process	4.8E-04	Regulation of programmed cell death	5.0E-18
	Blood circulation	4.8E-04	Regulation of cell death	6.5E-18
	Neuron projection development	6.5E-04	Protein transport	2.6E-17
	Cell motion	8.2E-04	Protein modification by small protein conjugation	2.9E-17
CC	Plasma membrane part	2.9E-04	Cytosol	1.4E-44
	Synapse	1.1E-03	Nuclear lumen	1.8E-40
	Cell junction	1.3E-03	Organelle lumen	4.0E-38
	Intrinsic to plasma membrane	2.6E-03	Membrane-enclosed lumen	3.0E-37
	Integral to plasma membrane	5.0E-03	Intracellular organelle lumen	1.1E-36
MF	Sequence-specific DNA binding	5.1E-04	Transcription factor binding	1.7E-21
	Transcription activator activity	3.3E-03	Identical protein binding	2.8E-20
	Enzyme inhibitor activity	3.8E-03	Enzyme binding	2.2E-15
	Transcription coactivator activity	5.1E-03	Small conjugating protein ligase activity	4.3E-14
	Transcription factor activity	1.2E-02	Protein C-terminus binding	1.1E-12

## Discussion

The determination of driver nodes that allow the control of underlying networks has attracted considerable attention in recent years. In particular, the MDS model has been applied to protein interaction networks to identify biologically central proteins. However, previous studies mainly focus on static protein interaction networks which lack tissue specificity, therefore their results may be inadequate. To overcome this shortcoming, we develop a corrected MDS model which picks up the MDS of which the members have the highest collective influence among all possible MDS configurations. We also construct 16 tissue-specific networks by integrating molecular expression profiles and static protein interaction maps. Then the developed new model is applied to the constructed tissue-specific networks to determine tissue dependent MDS proteins which are classified as TS-MDS proteins and HK-MDS proteins. We find that these two types of MDS proteins have different topological and functional properties, which shows the importance of tissue specificity for the study of the control of molecular interaction networks.

Several studies have, in fact, drawn attention to the problem of identifying real sets of driver proteins from multiple possible MDS configurations [12, 16]. Nacher and Akutsu [16] classified the nodes depending on the condition whether a node is part of all (critical), some but not all (intermittent), or does not participate in any (redundant) possible MDS. However, to obtain the classification of nodes, we need to solve the MDS model |*V*| times, where |*V*| is the number of nodes. Therefore, compared with computing an MDS, their method needs much more CPU time. Zhang et al. [12] proposed a Centrality-Corrected Minimum Dominating Set (CC-MDS) model which takes into account the degree and betweenness centralities of proteins. However, there is a weighting parameter in their model, and the authors suggested using a grid search method to determine parameter value. In doing so, we need to solve the CI-MDS model *K* times, where *K* is the number of considered values of weighting parameter. Unlike the two previously mentioned methods, our model only needs to solve the MDS model two times. Firstly, we need to solve the standard MDS model (Eq. 1) to compute the domination number (Eq. 2). Then, we need to solve the CI-MDS model (Eq. 4) to compute the MDS of which the members have the highest collective influence. In addition, the collective influence considered in the CI-MDS model is more effective in identifying powerful influencers than the degree and betweenness centralities considered in the CC-MDS model [7]. In particular, collective influence can uncover low-degree nodes surrounded by hierarchical coronas of high-degree nodes which may be neglected by the degree and betweenness centralities. Therefore, compared with the CC-MDS model, the CI-MDS model can discover more low degree proteins that play a major broker role in the network and have significantly functional roles. Note that the distant parameter *ℓ* in the CI-MDS model is different from the weighting parameter in the CC-MDS model. All possible values of distant parameter can produce valid MDS; while the weighting parameter needs to be tuned carefully to make sure the resulting set is a valid MDS.

Due to the development of high-throughput techniques such as yeast two-hybrid and co-immunoprecipitation [75, 76], a large number of physical interactions between proteins have been generated. Nevertheless, these interactions have rarely been characterized in the context of tissues because high-throughput interaction measurements are largely infeasible in solid tissues. While tissue-specific interactions are limited, molecular expression profiles across tissues have been rapidly accumulated [19–26]. Therefore, a data-driven approach can be used to identify tissue-specific interactions by integrating static physical interactions and tissue-specific expression profiles. There are two types of methods that convert a static interaction network into tissue-specific networks [36]: (1) node removal method which removes proteins which are not expressed in that tissue from the static network; (2) edge reweight method which modifies the edge weights to reflect the probability that the corresponding interactions occur in that tissue. In this study, we focus on the node removal method because the MDS model can only be applied to unweighted networks. The tissue-specific networks constructed using node removal method would depend on the stringent thresholds used to determine whether a protein is expressed in a tissue. Different thresholds may produce different networks. Here we set the thresholds following the method of [29, 42] and do not discuss how the thresholds influence the resulting networks.

Previous studies on tissue-specific networks mainly focus on comparing topological and functional features of tissue-specific proteins and housekeeping proteins. The tissue interactomes have also been applied to shed light on disease mechanisms. However, to the best of our knowledge, this study is a pioneer work that determines driver proteins in tissue-specific networks. Analogous to the definitions of tissue-specific proteins and housekeeping proteins [32], there are different criteria to define TS-MDS proteins and HK-MDS proteins. Following the method of Barshir et al. [42] which defines proteins expressed in 14 – 16 tissues as housekeeping proteins and proteins expressed in 1 – 3 tissues as tissue-specific proteins, we define proteins which are stated and identified as MDS proteins in at least 14 tissues as HK-MDS proteins and proteins which are expressed and selected as MDS proteins in at most 3 tissues as TS-MDS proteins. Comparative analysis reveals that the two types of MDS proteins exhibit significantly different functional characteristics. It is important to note that comparative experiment results may change with respect to the classification criteria. However, similar to the comparative analysis of tissue-specific proteins and housekeeping proteins, it would be expected that the comparative results would not change significantly.

## Conclusions

In this study, we construct 16 tissue-specific protein interaction networks by integrating tissue-specific expression profiles and static protein interactions. We also develop an extension of the standard Minimum Dominating Set (MDS) model and apply it to the constructed tissue-specific networks to identify MDS proteins (The detected MDS proteins are graphically visualized in Additional file 10). The identified MDS proteins are classified into tissue-specific MDS proteins and housekeeping MDS proteins. Through a comprehensive analysis, we find that the two types of MDS proteins exhibit significantly different topological and functional properties. These results suggest that tissue-specific networks will facilitate the discovery of driver proteins in human interactomes.

## Methods

### Datasets

#### Protein interaction network

Human binary protein interactions are extracted from the High-quality INTeractomes (HINT) database (version: 23 June 2015) [40]. Interactions in this database can be categorized into binary interactions and co-complex associations. Here we only consider binary interactions that represent direct physical contacts between proteins [77]. These interactions are collected from several databases and low-quality interactions are removed. Proteins are mapped to HUGO Gene Nomenclature Committee (HGNC) symbol identifiers [78], and proteins without known gene symbols are removed. The complete network consists of 56,695 interactions between 12,539 proteins.

#### Expression data

We use three expression profiles which are also used by Barshir et al. [42] to determine which interactions can occur in a particular tissue. A gene is considered to be expressed in a tissue if its expression value exceeds a stringent threshold. For detail, refer to [42]. In this study, we use the data provided in the MyProteinNet database [29].

#### Gene Ontology

Gene Ontology (GO) annotations of human proteins are obtained from the GO database (version: 20 August 2015) [54]. All the three domains (Biological Process (BP), Cellular Component (CC) and Molecular Function (MF)) are considered. Annotations with evidence code IEA, ND and NAS are excluded. We also do not consider annotations with NOT qualifier.

#### Evolutionary rate

We characterize the evolution rates of human proteins by calculating their dN/dS ratios. The synonymous and non-synonymous substitution rates between human and mouse are obtained from Ensembl (www.ensembl.org/biomart/martview/) (version: 19 August 2015) [58, 79].

#### Protein post-translation modifications

We retrieve the data for human Post-Translational Modifications (PTMs) from the dbPTM database (version: 23 August 2015) [61]. For each protein, the number of PTM sites are calculated.

#### Essential genes

A total of 2,501 human essential genes are collected from the Database of Essential Genes (DEG) (version: 19 August 2015) [62]. These data are retrieved from two studies that identify human essential genes using comparative genomics analysis [80, 81].

#### Aging genes

We collect 298 human ageing genes that are related to ageing from the Ageing Gene (GenAge) Database (version: 19 August 2015) [63].

#### Disease-associated genes

We retrieve 3,182 disease-related genes from the Online Mendelian Inheritance in Man (OMIM) database (version: 19 August 2015) [71]. In the “morbidmap” file, we do not consider disorders with symbols “[ ]”, “?”, “(1)”, “(2)”, “(4)”.

#### Cancer-related genes

We collect cancer-related genes from the Genome-Wide Association Studies (GWAS) Catalo database (version: 15 July 2016) [72]. Single-nucleotide polymorphism (SNP)-cancer associations with p-value less than 10^−5^ are considered, and the corresponding genes reported by authors are regarded as cancer-related genes. A total of 1,448 cancer-related genes are obtained.

#### Virus-targeted proteins

We obtain virus-host (human) protein interactions from the VirusMentha database (version: 19 August 2015) [65]. Proteins that interact with at least one virus protein are considered as virus-targeted proteins. A total of 2,420 virus-targeted proteins are obtained.

#### Transcription factors

We collect 222 human transcription factors from the TRANSFAC database [67] as provided by the MSigDB database [82] (version: 11 November 2014).

#### Protein kinases

We obtain 516 protein kinases in human from the Regulatory Network in Protein Phosphorylation (RegPhos) database (version: 2.0) [69].

For all datasets, we convert gene ID to HGNC gene symbols using BioMart [79], and we only consider proteins with known gene symbols in the experiments.

### Minimum dominating set model

A set *S*⊂*V* of nodes in a network *G*=(*V,E*) is considered to be a Dominating Set (DS) if every node *v*∈*V* is either an element of *S* or adjacent to an element of *S* [6, 14]. In other words, a DS is a subset of nodes from which all the remaining (e.g., non-DS) nodes can be reached by one step. A Minimum Dominating Set (MDS) is the smallest DS for a given network (Fig. 3). To determine an MDS, each node *v* is assigned with a binary integer variable *x*_*v*_, where *x*_*v*_=1 represents node *v* is an element of MDS and *x*_*v*_=0 otherwise. Mathematically, a DS needs to satisfy the following constraints $x_{v} + \sum _{u \in N(v)} x_{u} \ge 1$ for every node *v*, where *N*(*v*) is the set of neighbors of node *v*. Then the determination of an MDS that contains the fewest members among all DSs can be modeled as the following binary integer-programming problem:

1 $$ \left \{ \begin{array}{ll} \mathop{\text{minimize}}\limits_{x_{v} \in \{0,1\}} & \sum_{v \in V} x_{v} \\ \text{subject \ to} & x_{v} + \sum_{u \in N(v)} x_{u} \ge 1 \ \ \ \ \text{for all} \ v \in V. \\ \end{array} \right.   $$

This binary integer-programming problem is NP-complete, and the branch-and-bound algorithm is widely used to solve it [6, 83]. Here, we implement the algorithm using two softwares: library “lp_solve” of the MATLAB program language [43] and function “intlinprog” which is available in the Optimization ToolBox of MATLAB version R2014b [44]. We refer to this model as standard MDS model.

The domination number *γ*(*G*) of a network *G* is the number of nodes in an MDS. After obtaining an MDS by solving problem (1), we can calculate the domination number as follows:

2 $$ \gamma(G) = \sum_{v \in V} x_{v}.   $$

### Collective influence

Collective Influence (CI) is a newly developed centrality to quantify nodes’ influence in a network [7]. The collective influence of a node *v* is defined as the product of the node’s reduced degree (the number of neighbors minus one) and the sum of the reduced degrees of all nodes at distant *ℓ* from it:

3 $$ {\small{\begin{aligned} \text{CI}_{\ell} (v) = \left(d_{v} - 1\right) \sum_{u \in \partial \text{Ball} \left(v, \ell \right)} \left(d_{u} - 1\right),  \end{aligned}}}  $$

where *d*_*v*_ is the degree of node *v* and *∂*Ball(*v*,*ℓ*) represents the set of nodes that are *ℓ* hops away from node *v*. Collective influence quantifies how many other nodes can be reached from a given node. Therefore, we can assume that nodes with high collective influence play a crucial role in the entire network [8].

The collective-influence algorithm has a free parameter *ℓ* which needs to be determined. When *ℓ*=0, the collective influence of a node is equal to the square of its reduced degree, and it will perform in a similar way to degree centrality. To improve the performance, the authors [7] suggest choosing a non-zero but not too large *ℓ*. This is because that if *ℓ* is too large the boundaries of the network will be reached and the collective influence of all nodes approaches zero.

### Collective-influence-corrected minimum dominating set model

As mentioned in [12, 16], there may exist more than one optimal solution to the binary optimization problem (1) for a given network. Therefore, quite different MDS configurations may be produced using different optimization methods, and it is difficult to determine which one represents the real set of driver nodes.

To overcome this problem, we take into account the collective influence of nodes. Because nodes with higher collective influence are more likely to be drivers than nodes with low collective influence [7, 8], we would like to pick up the MDS of which the members have highest collective influence among all the MDS configurations (Fig. 3). We develop a Collective-Influence-corrected Minimum Dominating Set (CI-MDS) model as follows:

4 $$ \left \{ \begin{array}{ll} \mathop{\text{maximize}}\limits_{x_{v} \in \{0,1\}} & \sum_{v \in V} CI_{\ell}(v) \cdot x_{v} \\ \text{subject \ to} & x_{v} + \sum_{u \in N(v)} x_{u} \ge 1 \ \ \ \ \text{for all} \ v \in V, \\ & \sum_{v \in V} x_{v} = \gamma(G), \\ \end{array} \right.  $$

where *CI*_*ℓ*_(*v*) is the collect influence of node *v* (Eq. 3) and *γ*(*G*) is the domination number of graph *G* (Eq. 2). The constraint $ x_{v} + \sum _{u \in N(v)} x_{u} \ge 1$ ensures that the set is a DS, and the constraint $\sum _{v \in V} x_{v} = \gamma (G)$ ensures that the size of the set is equal to the domination number. Therefore, these two constraint ensure that the set is an MDS. The objective function $\sum _{v \in V} CI_{\ell }(v) \cdot x_{v}$ is used to identify nodes of highest collective influence.

Equation (4) is also a binary integer-programming problem, and can be solved using library “lp_solve” and function “intlinprog”. Before implementing the CI-MDS model (4), we need to determine an MDS using the standard MDS model (Eq. 1) and calculate the domination number *γ*(*G*) using Eq. (2). Because of collective influence term in the objective function, there is a free parameter *ℓ* in the CI-MDS model. We discuss the effect and choice of *ℓ* in the “Results” section.

### Definitions of tissue-specific and housekeeping MDS proteins

We construct 16 tissue-specific networks by combining three expression data (GNF, HPA and RNA-seq) with the global protein interaction network (Fig. 1). In particular, the global network is converted into a tissue-specific network by retaining only those interactions whose interacting partners are found to be expressed in that tissue according to at least one expression data. Then the CI-MDS model is applied to each tissue-specific network to determine tissue dependent MDS proteins. These identified MDS proteins are classified based on the number of tissues in which they are expressed and identified as MDS proteins (Fig. 1). Proteins that are expressed and identified as MDS proteins in at most 3 tissues are defined as Tissue-Specific MDS (TS-MDS) proteins. Proteins that are expressed and selected as MDS proteins in at least 14 tissues are defined as HouseKeeping MDS (HK-MDS) proteins.

### Biological functional enrichment analysis

We use DAVID for GO functional enrichment analysis of the sets of TS-MDS proteins and HK-MDS proteins [74].

All statistical tests employed in this study are implemented using MATLAB.

## Additional files

Additional file 1 Constructed tissue-specific protein interaction networks in 16 tissues. (XLSX 7067 kb)

Additional file 2 Overlap between MDSs computed using “lp_solve” and “intlinprog”. (XLSX 11 kb)

Additional file 3 Overlap between MDSs determined by the CI-MDS model with different distance parameter *ℓ*. (XLSX 14 kb)

Additional file 4 Different types of MDS proteins identified from tissue-specific networks. (XLSX 229 kb)

Additional file 5 Significance of the difference on node centralities between different types of proteins. (XLSX 10 kb)

Additional file 6 Significance of the difference on the number of associated GO terms between different types of proteins. (XLSX 10 kb)

Additional file 7 Distribution of the number of associated (A) biological process, (B) cellular component and (C) molecular function terms of different types of proteins. The distribution is represented by box plots (line = median). In each figure, outliers have been masked for clarity. Both direct GO annotations and all parent terms are taken into account. (EPS 2014 kb)

Additional file 8 Significance of the difference on evolutionary rates and the number of PTM sites between different types of proteins. (XLSX 9 kb)

Additional file 9 GO term enrichments for TS-MDS proteins and HK-MDS proteins. (XLSX 124 kb)

Additional file 10 MDS proteins (blue diamond nodes) and their associations with their neighbors for all 16 tissues. This is a Cytoscape file which can be opened using Cytoscape (version: 3.2.0). (CYS 18348 kb)

## Abbreviations

CI-MDS model: Collective-influence-corrected minimum dominating set model
GO: Gene ontology
HK-MDS proteins: Housekeeping MDS proteins
MDS: Minimum dominating set
TS-MDS proteins: Tissue-specific MDS proteins

## References

[1] Barabasi AL, Oltvai ZN (2004). Network biology: understanding the cell’s functional organization. Nat Rev Genet.

[2] Vinayagam A, Zirin J, Roesel C, Hu Y, Yilmazel B, Samsonova AA, Neumüller RA, Mohr SE, Perrimon N (2014). Integrating protein-protein interaction networks with phenotypes reveals signs of interactions. Nat Methods.

[3] Liu YY, Slotine JJ, Barabási AL (2011). Controllability of complex networks. Nature.

[4] Nacher JC, Akutsu T (2013). Structural controllability of unidirectional bipartite networks. Sci Rep.

[5] Ruths J, Ruths D (2014). Control profiles of complex networks. Science.

[6] Wuchty S (2014). Controllability in protein interaction networks. Proc Natl Acad Sci USA.

[7] Morone F, Makse HA (2015). Influence maximization in complex networks through optimal percolation. Nature.

[8] Kovács IA, Barabási AL (2015). Network science: Destruction perfected. Nature.

[9] Nacher JC, Akutsu T (2015). Structurally robust control of complex networks. Phys Rev E.

[10] Sun PG (2015). Controllability and modularity of complex networks. Inf Sci.

[11] Milenković T, Memišević V, Bonato A, Pržulj N (2011). Dominating biological networks. PLOS ONE.

[12] Zhang XF, Ou-Yang L, Zhu Y, Wu MY, Dai DQ (2015). Determining minimum set of driver nodes in protein-protein interaction networks. BMC Bioinforma.

[13] Khuri S, Wuchty S (2015). Essentiality and centrality in protein interaction networks revisited. BMC Bioinforma.

[14] Nacher JC, Akutsu T (2012). Dominating scale-free networks with variable scaling exponent: heterogeneous networks are not difficult to control. New J Phys.

[15] Hedetniemi ST, Laskar RC (1990). Bibliography on domination in graphs and some basic definitions of domination parameters. Discret Math.

[16] Nacher JC, Akutsu T (2014). Analysis of critical and redundant nodes in controlling directed and undirected complex networks using dominating sets. J Complex Netw.

[17] Gross AM, Ideker T (2015). Molecular networks in context. Nat Biotechnol.

[18] Yeger-Lotem E, Sharan R (2015). Human protein interaction networks across tissues and diseases. Front Genet.

[19] Santos A, Tsafou K, Stolte C, Pletscher-Frankild S, O’Donoghue SI, Jensen LJ (2015). Comprehensive comparison of large-scale tissue expression datasets. PeerJ.

[20] Su AI, Wiltshire T, Batalov S, Lapp H, Ching KA, Block D, Zhang J, Soden R, Hayakawa M, Kreiman G (2004). A gene atlas of the mouse and human protein-encoding transcriptomes. Proc Natl Acad Sci USA.

[21] Clark TA, Schweitzer AC, Chen TX, Staples MK, Lu G, Wang H, Williams A, Blume JE (2007). Discovery of tissue-specific exons using comprehensive human exon microarrays. Genome Biol.

[22] Fagerberg L, Hallström BM, Oksvold P, Kampf C, Djureinovic D, Odeberg J, Habuka M, Tahmasebpoor S, Danielsson A, Edlund K (2014). Analysis of the human tissue-specific expression by genome-wide integration of transcriptomics and antibody-based proteomics. Mol Cell Proteomics.

[23] Uhlén M, Fagerberg L, Hallström BM, Lindskog C, Oksvold P, Mardinoglu A, Sivertsson Å, Kampf C, Sjöstedt E, Asplund A (2015). Tissue-based map of the human proteome. Science.

[24] Berglund L, Björling E, Oksvold P, Fagerberg L, Asplund A, Szigyarto CA-K, Persson A, Ottosson J, Wernérus H, Nilsson P (2008). A genecentric human protein atlas for expression profiles based on antibodies. Mol Cell Proteomics.

[25] Kim MS, Pinto SM, Getnet D, Nirujogi RS, Manda SS, Chaerkady R, Madugundu AK, Kelkar DS, Isserlin R, Jain S (2014). A draft map of the human proteome. Nature.

[26] Wilhelm M, Schlegl J, Hahne H, Gholami AM, Lieberenz M, Savitski MM, Ziegler E, Butzmann L, Gessulat S, Marx H (2014). Mass-spectrometry-based draft of the human proteome. Nature.

[27] Barshir R, Basha O, Eluk A, Smoly IY, Lan A, Yeger-Lotem E (2013). The tissuenet database of human tissue protein–protein interactions. Nucleic Acids Res.

[28] Schaefer MH, Lopes TJ, Mah N, Shoemaker JE, Matsuoka Y, Fontaine JF, Louis-Jeune C, Eisfeld AJ, Neumann G, Perez-Iratxeta C (2013). Adding protein context to the human protein-protein interaction network to reveal meaningful interactions. PLoS Comput Biol.

[29] Basha O, Flom D, Barshir R, Smoly I, Tirman S, Yeger-Lotem E (2015). Myproteinnet: build up-to-date protein interaction networks for organisms, tissues and user-defined contexts. Nucleic Acids Res.

[30] Zhu F, Panwar B, Guan Y. Algorithms for modeling global and context-specific functional relationship networks. Brief Bioinform. 2015. doi:10.1093/bib/bbv065. 10.1093/bib/bbv065 PMC4945826 26254431

[31] Dezső Z, Nikolsky Y, Sviridov E, Shi W, Serebriyskaya T, Dosymbekov D, Bugrim A, Rakhmatulin E, Brennan RJ, Guryanov A (2008). A comprehensive functional analysis of tissue specificity of human gene expression. BMC Biol.

[32] Bossi A, Lehner B (2009). Tissue specificity and the human protein interaction network. Mol Syst Biol.

[33] Lin W-h, Liu W-c, Hwang M-j (2009). Topological and organizational properties of the products of house-keeping and tissue-specific genes in protein-protein interaction networks. BMC Syst Biol.

[34] Emig D, Albrecht M (2011). Tissue-specific proteins and functional implications. J Proteome Res.

[35] Guan Y, Gorenshteyn D, Burmeister M, Wong AK, Schimenti JC, Handel MA, Bult CJ, Hibbs MA, Troyanskaya OG (2012). Tissue-specific functional networks for prioritizing phenotype and disease genes. PLoS Comput Biol.

[36] Magger O, Waldman YY, Ruppin E, Sharan R (2012). Enhancing the prioritization of disease-causing genes through tissue specific protein interaction networks. PLoS Comput Biol.

[37] Ganegoda GU, Wang J, Wu FX, Li M (2014). Prediction of disease genes using tissue-specified gene-gene network. BMC Syst Biol.

[38] Greene CS, Krishnan A, Wong AK, Ricciotti E, Zelaya RA, Himmelstein DS, Zhang R, Hartmann BM, Zaslavsky E, Sealfon SC (2015). Understanding multicellular function and disease with human tissue-specific networks. Nat Genet.

[39] Kiran M, Nagarajaram HA (2013). Global versus local hubs in human protein–protein interaction network. J Proteome Res.

[40] Das J, Yu H (2012). Hint: High-quality protein interactomes and their applications in understanding human disease. BMC Sys Biol.

[41] Bradley RK, Merkin J, Lambert NJ, Burge CB (2012). Alternative splicing of rna triplets is often regulated and accelerates proteome evolution. PLOS Biol.

[42] Barshir R, Shwartz O, Smoly IY, Yeger-Lotem E (2014). Comparative analysis of human tissue interactomes reveals factors leading to tissue-specific manifestation of hereditary diseases. PLoS Comput Biol.

[43] lp_solve. http://lpsolve.sourceforge.net/5.5/. Accessed 16 Aug 2015.

[44] intlinprog. http://www.mathworks.com/help/optim/ug/intlinprog.html. Accessed 16 Aug 2015.

[45] Jeong H, Mason SP, Barabási AL, Oltvai ZN (2001). Lethality and centrality in protein networks. Nature.

[46] Song J, Singh M (2013). From hub proteins to hub modules: the relationship between essentiality and centrality in the yeast interactome at different scales of organization. PLoS Comput Biol.

[47] Freeman LC (1977). A set of measures of centrality based on betweenness. Sociometry.

[48] González AMM, Dalsgaard B, Olesen JM (2010). Centrality measures and the importance of generalist species in pollination networks. Ecol Complex.

[49] Joy MP, Brock A, Ingber DE, Huang S (2005). High-betweenness proteins in the yeast protein interaction network. BioMed Res Int.

[50] Becker E, Robisson B, Chapple CE, Guénoche A, Brun C (2012). Multifunctional proteins revealed by overlapping clustering in protein interaction network. Bioinformatics.

[51] Zhang XF, Dai DQ (2012). A framework for incorporating functional interrelationships into protein function prediction algorithms. IEEE/ACM Trans Computational Biol Bioinforma.

[52] Chapple CE, Robisson B, Spinelli L, Guien C, Becker E, Brun C (2015). Extreme multifunctional proteins identified from a human protein interaction network. Nat Commun.

[53] Pritykin Y, Ghersi D, Singh M (2015). Genome-wide detection and analysis of multifunctional genes. PLOS Comput Biol.

[54] Ashburner M, Ball CA, Blake JA, Botstein D, Butler H, Cherry JM, Davis AP, Dolinski K, Dwight SS, Eppig JT, Harris MA, Hill DP, Issel-Tarver L, Kasarskis A, Lewis S, Matese JC, Richardson JE, Ringwald M, Rubin GM, Sherlock G (2000). Gene ontology: tool for the unification of biology. Nat Genet.

[55] Liao BY, Scott NM, Zhang J (2006). Impacts of gene essentiality, expression pattern, and gene compactness on the evolutionary rate of mammalian proteins. Mol Biol Evol.

[56] Wang Z, Zhang J (2009). Why is the correlation between gene importance and gene evolutionary rate so weak. PLOS Genet.

[57] Fraser HB, Hirsh AE, Steinmetz LM, Scharfe C, Feldman MW (2002). Evolutionary rate in the protein interaction network. Science.

[58] Flicek P, Ahmed I, Amode MR, Barrell D, Beal K, Brent S, Carvalho-Silva D, Clapham P, Coates G, Fairley S (2013). Ensembl 2013. Nucleic Acids Res.

[59] Patil A, Kinoshita K, Nakamura H (2010). Hub promiscuity in protein-protein interaction networks. Int J Mol Sci.

[60] Duan G, Walther D (2015). The roles of post-translational modifications in the context of protein interaction networks. PLOS Comput Biol.

[61] Lee TY, Huang HD, Hung JH, Huang HY, Yang YS, Wang TH (2006). dbptm: an information repository of protein post-translational modification. Nucleic Acids Res.

[62] Zhang R, Lin Y (2009). Deg 5.0, a database of essential genes in both prokaryotes and eukaryotes. Nucleic Acids Res.

[63] Tacutu R, Craig T, Budovsky A, Wuttke D, Lehmann G, Taranukha D, Costa J, Fraifeld VE, de Magalhães JP (2013). Human ageing genomic resources: integrated databases and tools for the biology and genetics of ageing. Nucleic Acids Res.

[64] Gulbahce N, Yan H, Dricot A, Padi M, Byrdsong D, Franchi R, Lee DS, Rozenblatt-Rosen O, Mar JC, Calderwood MA, Baldwin A, Zhao B, Santhanam B, Braun P, Simonis N, Huh KW, Hellner K, Grace M, Chen A, Rubio R, Marto JA, Christakis NA, Kieff E, Roth FP, Roecklein-Canfield J, DeCaprio JA, Cusick ME, Quackenbush J, Hill DE, Munger K, Vidal M, Barabási AL (2012). Viral perturbations of host networks reflect disease etiology. PLOS Comput Biol.

[65] Calderone A, Licata L, Cesareni G (2014). Virusmentha: a new resource for virus-host protein interactions. Nucleic Acids Res.

[66] Spitz F, Furlong EE (2012). Transcription factors: from enhancer binding to developmental control. Nat Rev Genet.

[67] Matys V, Fricke E, Geffers R, Gößling E, Haubrock M, Hehl R, Hornischer K, Karas D, Kel AE, Kel-Margoulis OV, Kloos DU, Land S, Lewicki-Potapov B, Michael H, R. Münch IR, Rotert S, Saxel H, Scheer M, Thiele S, Wingender E (2003). Transfac®;: transcriptional regulation, from patterns to profiles. Nucleic Acids Res.

[68] Manning G, Whyte DB, Martinez R, Hunter T, Sudarsanam S (2002). The protein kinase complement of the human genome. Science.

[69] Huang KY, Wu HY, Chen YJ, Lu CT, Su MG, Hsieh YC, Tsai CM, Lin KI, Huang HD, Lee TY, Chen YJ (2014). Regphos 2.0: an updated resource to explore protein kinase–substrate phosphorylation networks in mammals. Database.

[70] Barabási AL, Gulbahce N, Loscalzo J (2011). Network medicine: a network-based approach to human disease. Nat Rev Genet.

[71] Hamosh A, Scott AF, Amberger JS, Bocchini CA, McKusick VA (2005). Online mendelian inheritance in man (omim), a knowledgebase of human genes and genetic disorders. Nucleic Acids Res.

[72] Welter D, MacArthur J, Morales J, Burdett T, Hall P, Junkins H, Klemm A, Flicek P, Manolio T, Hindorff L, Parkinson H (2014). The nhgri gwas catalog, a curated resource of snp-trait associations. Nucleic Acids Res.

[73] Weinstein JN, Collisson EA, Mills GB, Shaw KRM, Ozenberger BA, Ellrott K, Shmulevich I, Sander C, Stuart JM, Network CGAR (2013). The cancer genome atlas pan-cancer analysis project. Nat Genet.

[74] Huang DW, Sherman BT, Lempicki RA (2008). Systematic and integrative analysis of large gene lists using david bioinformatics resources. Nat Protoc.

[75] Havugimana PC, Hart GT, Nepusz T, Yang H, Turinsky AL, Li Z, Wang PI, Boutz DR, Fong V, Phanse S, Babu1 M, Craig SA, Hu P, Wan C, Vlasblom J, Dar V-u-N, Bezginov A, Clark GW, Wu GC, Wodak SJ, Tillier ERM, Paccanaro A, Marcotte EM, Emili A. A census of human soluble protein complexes. Cell. 2012; 150(5):1068–81. 10.1016/j.cell.2012.08.011 PMC3477804 22939629

[76] Rolland T, Taşan M, Charloteaux B, Pevzner SJ, Zhong Q, Sahni N, Yi S, Lemmens I, Fontanillo C, Mosca R, Kamburov A, Ghiassian SD, Yang X, Ghamsari L, Balcha D, Begg BE, Braun P, Brehme M, Broly MP, Carvunis AR, Convery-Zupan D, Corominas R, Coulombe-Huntington J, Dann E, Dreze M (2014). A proteome-scale map of the human interactome network. Cell.

[77] Zhang XF, Ou-Yang L, Hu X, Dai DQ (2015). Identifying binary protein-protein interactions from affinity purification mass spectrometry data. BMC Genomics.

[78] Gray KA, Yates B, Seal RL, Wright MW, Bruford EA (2014). Genenames.org: the hgnc resources in 2015. Nucleic Acids Res.

[79] Smedley D, Haider S, Durinck S, Pandini L, Provero P, Allen J, Arnaiz O, Awedh MH, Baldock R, Barbiera G (2015). The biomart community portal: an innovative alternative to large, centralized data repositories. Nucleic Acids Res.

[80] Liao BY, Zhang J (2008). Null mutations in human and mouse orthologs frequently result in different phenotypes. Proc Natl Acad Sci USA.

[81] Georgi B, Voight BF, Bućan M (2013). From mouse to human: evolutionary genomics analysis of human orthologs of essential genes. PLOS Genet.

[82] Liberzon A, Subramanian A, Pinchback R, Thorvaldsdóttir H, Tamayo P, Mesirov JP (2011). Molecular signatures database (msigdb) 3.0. Bioinformatics.

[83] Land AH, Doig AG (1960). An automatic method of solving discrete programming problems. Econometrica: J Econ Soc.

